# Switch Costs Occur at Lemma Stage When Bilinguals Name Digits: Evidence from Language-Switching and Event-Related Potentials

**DOI:** 10.3389/fpsyg.2016.01249

**Published:** 2016-08-31

**Authors:** Song Chang, Jiushu Xie, Li Li, Ruiming Wang, Ming Liu

**Affiliations:** ^1^Guangdong Provincial Key Laboratory of Mental Health and Cognitive Science, Center for Studies of Psychological Application, School of Psychology, South China Normal UniversityGuangzhou, China; ^2^College of International Culture, South China Normal UniversityGuangzhou, China

**Keywords:** bilingual, language production, switch costs, locus, ERP

## Abstract

Switch costs are generally found in language switching tasks. However, the locus where switch costs occur during bilingual language production remains unclear. Several studies that used a cued language-switching paradigm have attempted to investigate this question in bilingual language production, but researchers have not reached a consensus. Moreover, we are interested in where switch costs occur when language selection occurs after lemma activation. Previous studies have not investigated this question because most previous studies presented language cues before or along with the stimuli. Therefore, we used a modified cued language-switching paradigm with a combined event-related potentials (ERPs) technique to explore the locus of switch costs during bilingual language production. The cue and stimulus were separated and presented in two different presentation sequences in which Indonesian–Chinese bilingual speakers were instructed to name digits in their L1 or L2 according to the color of the cue. The ERPs related to the cue and stimulus for two presentation sequences were measured. In the stimulus-cue sequence, the analysis that was time-locked to cues revealed a reversed switch cost as early as 220 ms after the cue onset; furthermore, a switch cost was shown in L1 with a late stage post-cue onset. The results suggested that when language selection occurred after lemma activation, the switch costs mainly occurred at the lemma selection stage. In the cue-stimulus sequence, the analysis that was time-locked to cues did not reveal significant main effects of switching, whereas the analysis that was time-locked to digits yielded a switch cost, again indicating that switch costs mainly occurred at the lemma selection stage rather than at the language task schema competition stage. Overall, our results indicated that when bilinguals spoke digits aloud in the language switching task, switch costs mainly occurred at the lemma selection stage.

## Introduction

Bilingual speakers have to switch between their first language (L1) and second language (L2) considering the appropriateness of the context. However, bilingual speakers can effortlessly switch between their L1 and L2 and rarely generate errors (Poulisse and Bongaerts, 1994). This remarkable ability has prompted the question of how bilingual speakers manager their two languages in language switching.

One common task to investigate the underlying mechanism of bilingual language production is language switching (e.g., Meuter and Allport, 1999; Costa and Santesteban, 2004; Costa et al., 2006). In this task, bilingual speakers are usually instructed to switch between their L1 and L2 according to a cue when they named digits or pictures. Many previous studies have reported worse performance in switch trials (which used different languages to name two subsequent items) relative to performance in non-switch trials (or repeat trials, which used the same language to name two subsequent items; e.g., Costa and Santesteban, 2004; Christoffels et al., 2007; Verhoef et al., 2009; Guo et al., 2013). This phenomenon is known as switch costs. For example, Meuter and Allport (1999) found that when bilingual speakers switched between their L1 and L2 to name digits, the switch trials resulted in longer response times (RTs) compared with non-switch trials.

However, the locus where switch costs occur in bilingual language switching remains unclear. According to the Inhibitory Control (IC) model, switch costs may occur at two possible loci: the language task schema competition stage and the lemma selection stage (Green, 1998). The language task schema competition stage refers to the phase during which naming an object in L1 vs. L2 competes according to the external cue. The lemma selection stage is the process in which the activated candidate lemmas compete to be produced. Switch costs may occur during the language task schema competition stage (Green, 1998; Roelofs, 2003), the lemma selection stage (Green, 1998), or both stages (Green, 1998; Kroll et al., 2006; Abutalebi and Green, 2008).

Few studies have investigated the switch costs loci in bilingual language production because most previous studies presented language cues and stimuli simultaneously (usually by using the color of the stimuli to indicate the naming language; Jackson et al., 2001; Christoffels et al., 2007; Misra et al., 2012). Hence, it is difficult to distinguish the language task schema competition phase from the lemma selection phase. Recently, Guo et al. (2013) reported an ERP study that attempted to investigate the inhibition loci during trilingual word production by presenting cues that preceded the stimuli. Trilingual speakers named digits in one of their three languages according to a cue in the n-2 language repetition paradigm. The results revealed marginally significant n-2 repetition effects on cue-locked ERPs but significant n-2 repetition effects on stimulus-locked ERPs, suggesting that the switch costs mainly occurred at the lemma selection phase rather than the language task schema competition phase. However, the results differed from those of Verhoef et al. (2010), who found a more-negative amplitude for L2 in switch trials than in repeat trials with a 200–350 ms post-cue onset, implying that switch costs occur at the language task schema competition stage. Nonetheless, they merely analyzed cue-locked ERPs but not stimulus-locked ERPs. Thus, it remains unclear whether switch costs would have also occurred during the lemma selection stage in the study by Verhoef et al. (2010). Overall, researchers have not reached a consensus regarding the locus of switch costs in bilingual language production.

Furthermore, it remains unclear where switch costs would occur when candidate words in both languages have been activated before knowing which language to use. This is a very common situation in real life because bilingual speakers normally choose the intended language after situational object perception. Consider Chinese–English bilingual speakers, for example. Upon seeing a vase, the meaning of the object (i.e., semantic information) is first activated, and this activation spreads to the lemma level, activating the words “vase” and “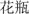 (hua ping)” in the bilingual speakers’ L1 and L2 (Green, 1998; Levelt et al., 1999). They then choose the target word to produce considering the appropriateness of the context. Thus, we are interested in where switch costs occur when the language task schema selection occurs after lemma activation. In specific, where is the switch costs mainly occur during bilingual language production when language selection occurs after the lemma activation: the language task schema competition stage or the lemma selection stage? This question has not been addressed in previous studies.

As mentioned above, most studies have presented language cues and stimuli simultaneously, thus binding the language task schema selection stage and lemma selection stage together (Jackson et al., 2001; Christoffels et al., 2007; Misra et al., 2012), or the studies presented cues that preceded the stimuli, thus causing the language schema selection stage to always occur before lemma selection (Levelt et al., 1999; Verhoef et al., 2009, 2010; Guo et al., 2013). These manipulations failed to investigate the specific process that occurs when language selection takes place after lemma activation in bilingual language production. Hence, in the current study, we used a modified cued language-switching paradigm in which the stimuli (digits: 1–8) were presented before the cues (colored squares: blue and red) to explore the specific switch costs locus when language selection occurs after lemma activation in bilingual language production.

Furthermore, as noted previously, researchers have not reached a consensus regarding the locus where switch costs occur in bilingual language production using the classical cue-stimulus sequence. Therefore, we also examined this debate by presenting cues that preceded the stimuli. By analyzing cue-locked and stimulus-locked ERPs, we expected to test the loci where switch costs occur during bilingual language production: the task schema competition stage, the lemma selection stage, or both stages.

## Materials and Methods

### Participants

Twenty Indonesian–Chinese bilingual speakers (Indonesians) from the International Culture College at South China Normal University (SCNU) participated in the current study. They were native Indonesian speakers who learned Chinese after the age of 10. The participants were asked to assess their language proficiency on a 7-point scale, with 7 indicating the highest level of fluency. Their average self-rated proficiency in Indonesian (L1) was 6.45 (*SD* = 0.65), compared with 4.66 (*SD* = 1.02) for Chinese (L2). They all had normal or corrected-to-normal vision. All participants provided written informed consent before the experiment and received a small monetary reward for their participation. The study was approved by the ethics review board of South China Normal University. Two participants’ data were eliminated because of excessive EEG artifacts. The EEG data from 18 participants (five males, 23.33 ± 4.21 years) were included in the final analysis.

### Materials and Procedure

In the current study, the bilingual speakers performed a digit-naming task in which they repeatedly used their L1 or L2 to name certain Arabic digits (i.e., 1–8). The naming language was determined by the color of a square (blue or red), which served as the language cue. Blue and red squares were presented pseudo-randomly so that subsequent trials could require the use of either the same language or a different one. According to the relationship of the two successive trials, we obtained four different language combinations: L1 non-switch trials, L1–L2 switch trials, L2–L1 switch trials, and L2 non-switch trials. Each combination included approximately 60 trials.

**FIGURE 1:**
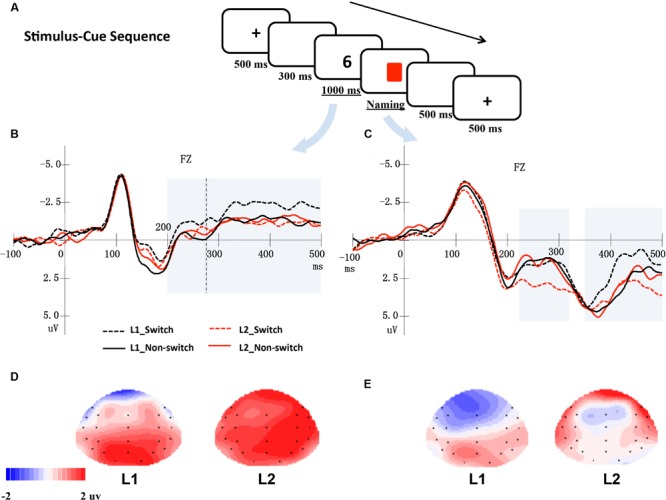
**(A)** An example of a trial in a stimulus-cue sequence. **(B)** Shows the averaged ERPs at electrode Fz elicited by digits, while **(C)** shows the ERPs at Fz elicited by cues. The shaded areas indicate windows of analysis. **(D,E)** Show the topographical maps for the distribution of the difference between switch and non-switch conditions for L1 and L2 based on cue-locked ERPs in the 220–320 ms **(D)** and 350–500 ms time windows **(E)**, respectively.

**FIGURE 2:**
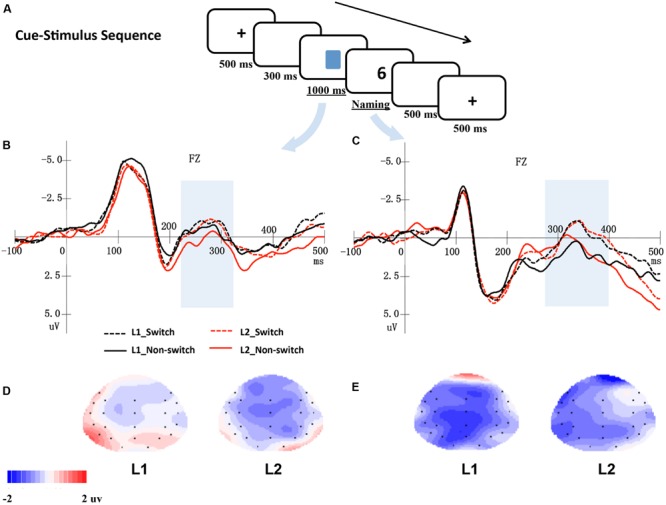
**(A)** An example of a trial in a cue-stimulus sequence. **(B)** Shows the averaged ERPs at electrode Fz elicited by cues, and **(C)** shows the ERPs at electrode Fz elicited by digits. The shaded areas indicate windows of analysis. The bottom panel presents topographical maps for the distribution of the difference between switch and non-switch conditions for L1 and L2 based on cue-locked ERPs **(D)** and stimulus-locked ERPs **(E)**, respectively.

As **Figures 1** and **2** show, there were two types of presentation sequences. In the first type of presentation sequence, the targets (i.e., digits) appeared earlier than the cues (i.e., colored squares). In this sequence type, each trial began with a 500 ms fixation, followed by a blank screen for 300 ms. Afterward, a digit was presented for 1000 ms at the center of the screen. A cue was then presented, and the trial waited for the participants’ responses. The participants were required to name the digit as quickly and accurately as possible in their L1 or L2, according to the cue. There was a 500 ms interval between the trials. In the second type of presentation sequence, the cues appeared earlier than the stimuli. In each trial, after a 500 ms fixation and a 300 ms blank screen, a language cue was presented for 1000 ms. A target digit was then presented at the center of the screen and waited for the participants’ responses. The experiment included two blocks. One block adopted the stimulus-cue presentation sequence, and the other block adopted the cue-stimulus presentation sequence. The participants completed both blocks. The order of the two blocks was counterbalanced across participants. The mapping of the square’s color and response language was also counterbalanced across participants. The experiment consisted of 480 critical trials.

### Electrophysiological Recording and Analyses

ERPs were continuously sampled at 1000 Hz and were band-pass filtered between 0.05 and 70 Hz from a 32-channel Quik cap (NeuroScan Inc.) that was referenced online to the left mastoid. Thirty-three Ag/AgCl electrodes were placed according to the 10–20 system. In addition, horizontal and vertical electro-oculograms (EOGs) were recorded to allow for artifact rejection in the analyses. The impedances of all electrodes were kept below 10 kΩ. ERPs were digitally filtered at a low pass of 30 Hz (24 dB setting). Given that the mean latencies for digit naming were longer than 500 ms after the onset of the stimuli, we adopted epochs that ranged from -100 to 500 ms after the stimulus onset to prevent the muscle artifact from being induced by naming. Epochs with voltages exceeding ±100 μV were treated as artifacts and were rejected. Only trials that were free from eye and muscle artifacts were included in the final analyses. There were no fewer than 30 trials in each condition on individual average. The individual ERPs were then grand-averaged for the presentation of results.

In the stimulus-cue sequence, digits were first presented for 1000 ms, which was sufficient to allow the participants to activate the representations of digits in two languages. Upon the cue presentation, the participants knew the naming language. Thus, bilingual language production processing was evoked, and it was possible to examine whether the bilingual speakers inhibited the non-target language first or retrieved the target language directly when both languages have already been activated. Therefore, we mainly focused on the cue-locked ERPs in the stimulus-cue sequence. Based on a visual inspection and previous studies (Christoffels et al., 2007; Verhoef et al., 2010; Guo et al., 2013), two time windows were selected for stimulus-locked analyses: 200–270 ms and 270–500 ms post-stimulus onset, and another two time windows were selected for cue-locked analyses: 220–320 ms and 350–500 ms. In the cue-stimulus sequence, the time window of 220–320 ms post-cue (i.e., colored squares) onset was chosen for the cue-locked analysis, and a time window of 270–400 ms post-stimulus (i.e., digits) onset was selected for the stimulus-locked ERPs analysis. Separate analyses were conducted for the lateral and midline electrode sites. Twenty-two electrodes (F3/4, FT7/8, FC3/4, F7/8, C3/4, TP7/8, CP3/4, P7/8, P3/4, O1/2, and FP1/2) were chosen as lateral electrode sites. A 2 (language: Indonesian or Chinese) ^∗^ 2 (language transition: switch or non-switch) ^∗^ 2 (hemisphere: left or right) ^∗^ 11 (electrodes) repeated-measure ANOVA was performed for the mean amplitudes of the ERPs in each time window. Furthermore, six midline electrodes (Fz, FCz, Cz, CPz, Pz, and Oz) were submitted to a 2 (language: Indonesian or Chinese) ^∗^ 2 (language transition: switch or non-switch) ^∗^ 6 (electrodes) ANOVA in each time window. Geisser-Greenhouse corrections were reported where applicable. Only statistically significant main effects of language transitions and related interactions were reported.

## Results

### Behavioral Data

**FIGURE 3:**
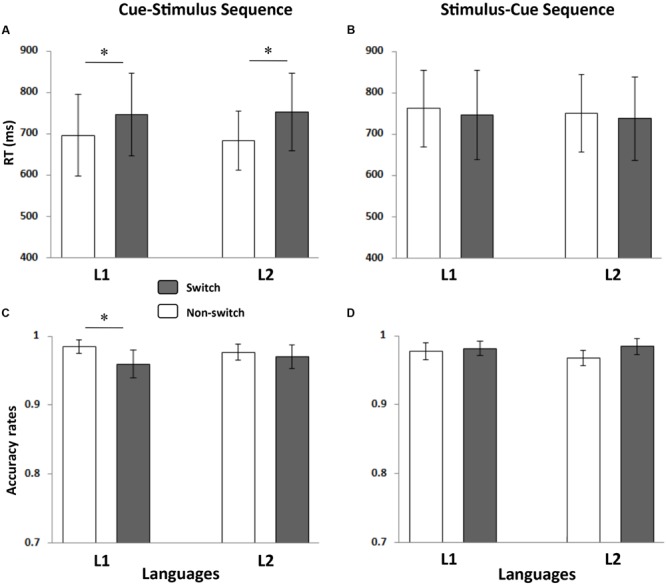
**Mean RTs and ACCs for each condition.** The left panel shows the mean RT **(A)** and ACC **(C)** of the cue-stimulus sequence, and the right panel presents the mean RT **(B)** and ACC **(D)** of the stimulus-cue sequence. The stars indicate a significant difference (*p* < 0.05). The error bars refer to standard errors (SE).

Three types of responses were excluded from the reaction time (RT) analyses: (i) incorrect naming, (ii) verbal dysfluencies, and (iii) very fast (shorter than 200 ms) and very slow responses (longer than 2000 ms). Trimming resulted in the removal of 2.38% of the data. All trials were included for accuracy (ACC) analyses. The mean RT and ACC were then calculated for each of the eight conditions and were summarized in **Figure 3**.

We performed a 2 (language: Indonesian or Chinese) ^∗^ 2 (language transition: switch or non-switch) ANOVA for the RTs and ACCs across both presentation sequences.

#### Stimulus-Cue Sequence

There were no significant main effects or interactions in the RT analysis. In the ACC analysis, only the main effect of the language transition was marginally significant, *F*(1,17) = 3.48, *p* = 0.079, $\eta_{p}^{2}$ = 0.170. Neither the main effect of language nor the interaction effect reached significance (*p*s > 0.1).

#### Cue-Stimulus Sequence

The RT analysis revealed that the main effect of the language transition was significant, *F*(1,17) = 46.561, *p* < 0.001, $\eta_{p}^{2}$ = 0.733, indicating that naming latencies were longer in the switch trials than in the non-switch trials. Neither the main effect of language nor the interaction effect was significant (*p*s > 0.1).

The ANOVA performed on ACCs showed that the language ^∗^ language transition interaction was significant, *F*(1,17) = 4.61, *p* = 0.047, $\eta_{p}^{2}$ = 0.213. Further simple effect analysis revealed that the participants made more errors in the switch trials than in the non-switch trials when they named the digits in L1, *F*(1,17) = 15.33, *p* = 0.001, but not in L2 (*p* > 0.1).

### Electrophysiological Data

In **Figures 1** and **2**, the grand mean waveforms for each condition are displayed separately for cue and digit. All conditions elicited a negative peak at approximately 100 ms followed by a positive peak at approximately 200 ms. The waveforms consisted of the N1 and P2 components, a typical pattern elicited by visual stimuli.

#### Stimulus-Cue Sequence

In this presentation sequence, digits elicited a negative-going wave, which began around 200 ms and lasted until 500 ms after stimuli onset. For cue-locked grand average waves, there was a negative-going component that peaked at approximately 280 ms and lasted approximately 100 ms, followed by a late negative-going component that peaked at approximately 450 ms after the onset of the stimulus in cue-locked grand average waveforms.

##### Digit-locked electrophysiological data (time window 200–270 ms)

The ANOVA performed on midline electrodes and lateral electrodes did not reveal any significant main effects of language and language transition or interactions (*p*s > 0.05).

##### Digit-locked electrophysiological data (time window 270–500 ms)

The ANOVA conducted for the midline electrodes and lateral electrodes did not reveal any significant main effects of language and language transition or interactions (*p*s > 0.05).

##### Cue-locked electrophysiological data (time window 220–320 ms)

The analysis of the midline electrodes revealed a significant main effect of the language transition, *F*(1,17) = 6.01, *p* = 0.025, $\eta_{p}^{2}$ = 0.261, reflecting that the ERPs were more negative for the non-switch trials than for the switch trials. Moreover, a significant interaction effect between language transition and electrodes was found, *F*(1,17) = 4.61, *p* = 0.024, $\eta_{p}^{2}$ = 0.213, with simple effect tests indicating that the mean amplitude of the non-switch trials was more negative than the switch trials over electrodes Pz, Oz, CPz and Cz (*p*s < 0.05).

The ANOVA conducted over the lateral electrodes showed that the interaction of language, language transition, and electrodes was significant, *F*(1,17) = 3.82, *p* = 0.048, $\eta_{p}^{2}$ = 0.184. Further simple effect tests revealed that when participants named the digits in L2, the non-switch trials elicited more negative ERPs than the switch trials across all electrodes (*p*s < 0.05) except Fp1 and Fp2 (*p*s > 0.05); however, this difference was not significant for most electrodes when participants named the digits in L1 (*p*s > 0.05).

##### Cue-locked electrophysiological data (time window 350–500 ms)

The ANOVA conducted for the midline electrodes did not reveal any significant effects.

According to the analysis of the lateral electrodes, there was a significant interaction effect of language, language transition, and electrodes, *F*(1,17) = 4.09, *p* = 0.040, $\eta_{p}^{2}$ = 0.194. Further simple effect analysis revealed that the mean amplitude elicited by the L1 switch trials was more negative than that elicited by the L1 non-switch trials over the frontal electrodes F7/8 (*p* = 0.038), F3/4 (*p* = 0.039), FT7/8 (*p* = 0.069), whereas there was no significant difference between the L2 switch and L2 non-switch trials.

#### Cue-Stimulus Sequence

In this sequence, there was a negative-going wave that peaked at approximately 280 ms and lasted approximately 100 ms in the cue-locked grand average waveforms. Meanwhile, a P2 followed by a large negative-going wave peaking at approximately 350 ms was observed in the digit-locked ERPs.

##### Cue-locked electrophysiological data (time window 220–320 ms)

The ANOVA of the data from the midline and lateral electrodes showed no significant main effects of language and language transition or interactions (*p*s > 0.05).

##### Digit-locked electrophysiological data (time window 270–400 ms)

According to the ANOVA of the data from the midline electrodes, there was a significant main effect of the language transition, *F*(1,17) = 7.35, *p* = 0.015, $\eta_{p}^{2}$ = 0.302, reflecting that the mean amplitude for the switch trials was more negative than that for the non-switch trials.

The ANOVA of the data from the lateral electrodes found a significant main effect of language transition, *F*(1,17) = 5.37, *p* = 0.033, $\eta_{p}^{2}$ = 0.240, indicating that the amplitude for switch conditions was more negative than that of the non-switch conditions. Furthermore, the interaction between language transition and hemisphere was significant, *F*(1,17) = 7.63, *p* = 0.013, $\eta_{p}^{2}$ = 0.310, suggesting that the ERPs of the switch trials were more negative than those of the non-switch trials in the left hemisphere, *F*(1,17) = 9.04, *p* = 0.008; however, no significant difference was found for the right hemisphere (*p*s > 0.05).

## Discussion

The present study used modified cued language-switching tasks to investigate the locus of switch costs during bilingual word production. We separated the cue and stimulus and presented them in either a stimulus-cue sequence or a cue-stimulus sequence. By analyzing the cue-locked and stimulus-locked ERPs data from these two presentation sequences, we investigated the locus where switch costs occur when bilinguals name digits. In the stimulus-cue sequence, the analysis time-locked to cues revealed a reversed switch cost as early as 220 ms after the cue onset, whereas a switch cost in L1 occurred after 350–500 ms post-cue onset, suggesting that the switch costs mainly occurred at the lemma selection stage. In the cue-stimulus sequence, the ERPs that were time-locked to digits yielded a switch cost, whereas the ERPs that were time-locked to cues did not reveal a significant main effect of the switch. The results indicated that switch costs mainly occurred at the lemma selection stage when bilinguals read digits aloud in the language switching task.

In this study, the modified language-switching paradigm, which presented stimuli that preceded cues, allowed us to investigate the specific processing that takes place when language selection occurs after lemma activation during bilingual language production. Upon digit presentation, candidate words in the participants’ L1 and L2 were activated. When the cues were then shown, the candidate words competed to be produced according to the cue. We were interested in where the switch costs primarily occurred when language selection occurred after the lemma activation. The analysis that was time-locked to stimuli did not reveal any differences among the naming conditions. However, it was possible that participants were biased toward switch trials in this unpredictable switch task. This expectation effect would cause greater activation of the digit name in L1 or L2 according to the preceding trial. Actually, it is impossible to know the participants’ actual expectations. Thus the difference between activation of L1 and L2 is hard to detect based on stimulus-locked ERPs. Alternatively, it seems that this difference caused by expectation could be detected by the following cue-locked ERPs.

The analysis that was time-locked to cues revealed a fronto-central negative-going wave that began approximately 220 ms after cue onset, a finding that was similar to the N2 component reported in previous ERP studies on bilingual language production (Jackson et al., 2001; Christoffels et al., 2007; Guo et al., 2013). However, the non-switch trials elicited more negative-going amplitudes than the switch trials did, indicating a reversed switch cost. In contrast, the cue-locked ERP results revealed a switch cost in the time window of 350–500 ms after cue-onset when naming the digits in L1 but not in L2. The negative-going ERP that peaked approximately 450 ms after the cue onset may be N400, which reflected the lemma retrieval process. According to the IC model, lemma selection happens after task schema competition in bilingual language production. Therefore, it seems that the former negative ERP component corresponds to the language task schema competition stage and the late negative ERP component corresponds to the lemma selection stage. Thus, the results in the stimulus-cue sequence indicated that switch costs mainly occured at the lemma selection stage when bilinguals name digits.

It should be noted that regarding the switch cost, we found a reversed effect in the time window of 220–320 ms after the cue-onset. Christoffels et al. (2007) found a similar reversed switch cost, in which repeat trials elicited greater N2 amplitudes than switch trials. In that study, moderately proficient German–Dutch bilingual speakers were asked to name pictures while switching between their L1 and L2 (mixed language condition; Christoffels et al., 2007). In comparison, Jackson et al. (2001) used a similar language-switching paradigm and found a normal switch cost, in which the switch trials elicited more-negative N2 compared with non-switch trials. One of the important differences between these two studies was the predictability of the language switch. Specifically, the language of the subsequent trial was unpredictable in the study by Christoffels et al. (2007) but was fully predictable in the study by Jackson et al. (2001). Because of the unpredictability of the language switch, the bilingual speakers may have biased toward switching to help them respond in the mixed language context. Christoffels et al. (2007) assumed that because of the bias, non-switch trials would elicit greater conflict than switch trials that was reflected by the enlarged N2 amplitude. This tentative explanation could also account for the results of the present study, in which the language used to name digits was unpredictable. For example, if participants named the digit in L1 in the preceding trial, they might expect to name the next trial in L2 (i.e., switch trials). Thus upon digit presentation, the digit’s name in L2 received more activation than its name in L1. However, if the next trial still required the participants to name digits in L1 (i.e., non-switch trials), the activation of digit’s name in L2 would be inhibited, which was reflected by the N2 of cue-locked ERPs. Hence, the non-switch trials showed more conflict than the switch trials and elicited enhanced N2 negativity.

Furthermore, in the stimulus-cue sequence, we found a switch cost in the time window 350–500 ms after cue-onset when participants named in L1 but not in L2. One possible reason for the asymmetric switch cost may be bilingual speakers’ bias for preparing the words in L2. As we mentioned above, bilingual speakers have a bias for preparing words in their weaker L2 to facilitate naming. Thus, the words in L2 had similar or even higher activation levels than that in L1. Therefore, after selecting the target language task schema, bilingual speakers must exert some effort to control the activated L2 lemmas when switching from L2 to L1, thus eliciting enlarged N400 in L1 switch trials compared with L1 non-switch trials. In contrast, when naming in L2, L2 lemmas had relatively high activation levels regardless of whether a switch or repeat trial occurred. Thus, there was no difference between the L2 switch trials and the L2 non-switch trials.

The results of the cue-stimulus sequence provide converging evidence that switch costs mainly occur at the lemma selection stage when bilinguals read digits aloud. In the time-window 220–320 ms, the ERPs that were time-locked to cues did not reveal significant main effects of switching. However, in the time-window 270–400 ms, the ERPs that were time-locked to digits yielded a significant switch cost. Again, the results indicated that when bilinguals read digits aloud, switch costs mainly occurred at the lemma selection stage rather than at the language task schema competition stage. This result is consistent with Guo et al.’s (2013) findings, which suggested that switch costs were involved in the lemma selection phase.

However, our results differ from Verhoef et al.’s (2010) findings of more negative cue-locked ERPs for switch trials than for non-switch trials, which suggested that switch costs were involved in the language task schema competition phase. A similar cued language-switching task and unbalanced bilinguals were used in both studies. We assume that different typologies of L1 and L2 of bilingual speakers may account for the varying results of Verhoef et al. (2010). Specifically, in the study by Verhoef et al. (2010), the participants’ L1 and L2 were Dutch and English, which both used alphabetic scripts and were similar to each other in many aspects. In contrast, in the study by Guo et al. (2013) and the present study, L1 and L2 of participants differ greatly: L1 used an alphabetic script, whereas L2 (i.e., Chinese) used an ideographic script. Previous studies found that compared to bilinguals whose two languages have a less degree of orthographic overlap, bilinguals whose two languages have larger overlaps would require greater cognitive control to manage their languages (Coderre and van Heuven, 2014). It is possible that the small linguistic distance between the participants’ two languages in the study by Verhoef et al. (2010) may increase control of the non-target language. Thus, in their study switch costs might also occur at the language task schema competition phase.

In the cue-stimulus sequence, the behavioral data did not indicate an asymmetric switch cost between the dominant L1 and the less-dominant L2. The RTs in our study were longer in the switch trials than in the non-switch trials, independent of the response language. These results differ from those of previous studies that found asymmetric switch costs when unbalanced bilingual speakers performed switched naming tasks (Meuter and Allport, 1999; Costa and Santesteban, 2004; Philipp et al., 2007). The cued language-switching task used here may account for these differences. In the present study, the cue was presented 1000 ms before the digits, and the relatively long preparation time may have reduced the asymmetric effect induced by the differences in language proficiency. In fact, some studies using cued naming tasks also found that when the cue preceded the stimulus by a relatively long interval, the asymmetric switch costs disappeared (Verhoef et al., 2009, 2010; Guo et al., 2013).

Moreover, in the stimulus-cue sequence, there was no significant difference between the switch and repeat trials in RTs, which failed to reveal a switch cost. This result might be because, in the stimulus-cue sequence, the digits appeared for 1000 ms before the cue, thus the bilingual speakers had sufficient time to activate the candidate words in L1 and L2 before production. In contrast, the time allotted for digit processing was shorter in the cue-stimulus sequence. It seems that when bilingual speakers have sufficient time to access the candidate lemmas, the switch costs could disappear. In this sense, our behavioral data also lend some support to the notion that switch costs mainly involved in the lemma selection phase.

Previous findings showed that bilinguals could restrict to the target language and reduce interference from the non-target language through cognitive control during language switching. Several cognitive models have attempted to explain the control mechanism underlying bilingual language processing (Costa, 2005; La Heij, 2005; Finkbeiner et al., 2006). Some language control models rely on inhibitory mechanisms (e.g., IC model), whereas some language control models do not rely on inhibition. For example, some researchers proposed the interference account for switch costs in bilingual language production, assuming that the persistent activation of the language in naming the previous trial has certain influences on the following trial, resulting in switch costs (Philipp et al., 2007). Some efforts have been made to differentiate between inhibition and interference during bilingual/trilingual language production. For example, previous studies adopted an independent-probe test and the n-2 task repetition paradigm, which allow for direct test to differentiate between inhibition and interference, suggesting that inhibitory control, but not interference, was involved in bilingual/trilingual language production (Levy et al., 2007; Philipp and Koch, 2009; Koch et al., 2010; Guo et al., 2013). It seems that switch costs observed in the current study may also reflect an inhibitory control process. However, the paradigm used here differs from previous studies. Whether our results reflect inhibition needs further investigation.

Both digit-naming task and picture-naming task are widely used in bilingual language production studies. We chose to use numerical digits as stimuli in the current study primarily based on the following considerations. First, compared to pictures, digits are more consistent in name agreement and easier to control for phonological and semantic influences. Second, the same digits (e.g., 1–9) are usually used in digit-naming tasks and this manipulation makes comparisons across studies possible. However, it may be argued that the limited digits have potential influences on pre-stimulus control processing. Specifically, in real-life speech production, pre-stimulus language control may involve the inhibition of the entire non-target language system and the preparation for the target language system. In the digit-naming task, this process may be simplified to the suppression of a set of digits in the non-target language and the preparation of these digits in the target language. Naming the certain set of digits in a language switching task might not elicit cognitive control at the language task schema competition phase. However, no study has investigated the influences of stimulus type on pre-stimulus cognitive control during bilingual language production to date. A previous study showed that when cognate status of digits and pictures were controlled, no switch costs difference was found between them, which implies that when phonology is controlled for, stimulus type (i.e., pictures and digits) may have little influence on bilingual switch costs (Declerck et al., 2012). Whether stimulus type influences pre-stimulus language control needs further investigation.

## Conclusion

The current study investigated the locus of switch costs during bilingual word production. The main findings of the present study indicated that when bilinguals read digits aloud in the language switching task, switch costs mainly occurred at the lemma selection stage rather than at the language task schema competition stage.

## Author Contributions

SC, LL, and RW designed the experiments. SC and JX ran the data-collection procedures of the experiments. SC, JX, and RW analyzed and interpreted the data. SC drafted the manuscript. JX, RW, and ML provided critical revisions of the manuscript. SC and JX contributed equally to this work.

## Conflict of Interest Statement

The authors declare that the research was conducted in the absence of any commercial or financial relationships that could be construed as a potential conflict of interest.
